# Reversal of cognitive decline in Alzheimer's disease

**DOI:** 10.18632/aging.100981

**Published:** 2016-06-12

**Authors:** Dale E. Bredesen, Edwin C. Amos, Jonathan Canick, Mary Ackerley, Cyrus Raji, Milan Fiala, Jamila Ahdidan

**Affiliations:** ^1^ Easton Laboratories for Neurodegenerative Disease Research, Department of Neurology, University of California, Los Angeles, CA 90095, USA; ^2^ Buck Institute for Research on Aging, Novato, CA 94945, USA; ^3^ Department of Neurology, University of California, Los Angeles, CA 90095, USA; ^4^ Memory Clinic, California Pacific Medical Center, San Francisco, CA 94115, USA; ^5^ Private Practice of Psychiatry, Tucson, AZ 85718, USA; ^6^ Department of Radiology, University of California, Los Angeles, CA 90095, USA; ^7^ Department of Surgery, University of California, Los Angeles, CA 90095, USA; ^8^ Brainreader, Horsens, Denmark

**Keywords:** neurodegeneration, cognition, biomarkers, dementia, neuropsychology, imaging, Alzheimer's disease, Apolipoprotein E

## Abstract

Alzheimer's disease is one of the most significant healthcare problems nationally and globally. Recently, the first description of the reversal of cognitive decline in patients with early Alzheimer's disease or its precursors, MCI (mild cognitive impairment) and SCI (subjective cognitive impairment), was published [1]. The therapeutic approach used was programmatic and personalized rather than monotherapeutic and invariant, and was dubbed metabolic enhancement for neurodegeneration (MEND). Patients who had had to discontinue work were able to return to work, and those struggling at work were able to improve their performance. The patients, their spouses, and their co-workers all reported clear improvements. Here we report the results from quantitative MRI and neuropsychological testing in ten patients with cognitive decline, nine ApoE4+ (five homozygous and four heterozygous) and one ApoE4−, who were treated with the MEND protocol for 5-24 months. The magnitude of the improvement is unprecedented, providing additional objective evidence that this programmatic approach to cognitive decline is highly effective. These results have far-reaching implications for the treatment of Alzheimer's disease, MCI, and SCI; for personalized programs that may enhance pharmaceutical efficacy; and for personal identification of ApoE genotype.

## INTRODUCTION

Alzheimer's disease is now the third leading cause of death in the United States, following only cardio-vascular disease and cancer [1]. There are approximately 5.2 million Americans with AD, but this estimate ignores the many young Americans destined to develop AD during their lifetimes: given the lifetime risk of approximately 15% when including all ApoE genotypes, as many as 45 million of the 318 million Americans now living may develop AD during their lifetimes if no prevention is instituted [2].

Effective treatment of Alzheimer's disease has been lacking, but recently a novel programmatic approach involving metabolic enhancement was described, with promising anecdotal results [3]. This treatment is based on connectomic studies [4] and previous transgenic findings [5] as well as epidemiological studies of various monotherapeutic components of the overall program [6]. The approach is personalized, responsive to suboptimal metabolic parameters that reflect a network imbalance in synaptic establishment and maintenance vs. reorganization, and progressive in that continued optimization is sought through iterative treatment and metabolic characterization.

Here we report the initial follow-up of ten patients who were treated with this metabolic programmatics approach. One patient had well documented mild cognitive impairment (MCI), with a strongly positive amyloid-PET (positron emission tomography) scan, positive FDG-PET scan (fluorodeoxyglucose PET scan), abnormal neuropsychological testing, and hippocampal volume reduced to 17th percentile; after 10 months on the MEND protocol, his hippocampal volume had increased to 75th percentile, in association with a reversal of cognitive decline. Another patient had well documented early Alzheimer's disease, with a positive FDG-PET scan and markedly abnormal neuro-psychological testing. After 22 months on the MEND protocol, he showed marked improvement in his neuropsychological testing, with some improvements reaching three standard deviations from his earlier testing.

The initial results for these patients show greater improvements than have been reported for other patients treated for Alzheimer's disease. The results provide further support for the suggestion that such a comprehensive approach [3] to treat early Alzheimer's disease and its precursors, MCI and SCI, is effective. The results also support the need for a large-scale, personalized clinical trial using this protocol.

## RESULTS

### Case studies

#### Patient 1

A 66-year-old professional man presented with what he described as “senior moments” (for example, forgetting where his keys were or forgetting appointments) of two-years duration, and difficulty performing his work. There was a positive family history of dementia in both parents. He was an ApoE4 heterozygote (3/4), his amyloid PET scan was markedly positive, and his fluorodeoxyglucose (FDG) PET scan showed temporoparietal reduced glucose utilization indicative of Alzheimer's disease. An MRI showed hippocampal volume at only 17^th^ percentile for his age. His neuropsychological testing was compatible with a diagnosis of MCI. His hs-CRP was 9.9mg/l, albumin: globulin ratio was 1.6, homocysteine 15.1μmol/l, fasting glucose 96mg/dl, hemoglobin A1c 5.5%, fasting insulin 32mIU/l, 25-hydroxychole-calciferol 21ng/ml, TSH 2.21mIU/l, and testosterone 264ng/dl.

He began the MEND protocol [3], lost 18 pounds, and after three months his wife reported that his memory had improved. He noted that his work came more easily to him. However, after five months, he discontinued the majority of the program for approximately three weeks. His wife came home to find his car in the driveway, idling with the keys in the ignition, while he was inside the house, working and unaware that he had left the car idling in the driveway. He re-initiated the program, and had no further such episodes.

After 10 months on the program, he returned for a follow-up MRI, which was subjected to volumetric analyses by both Neuroquant [7] and Neuroreader [8] programs. The former indicated an increase in hippocampal volume from 17^th^ percentile to 75^th^ percentile, with an associated absolute increase in hippocampal volume of 11.7%. The Neuroreader program showed an absolute increase from 7.65cc to 8.3cc, which represents an 8.5% absolute increase in size. The associated Z-scores were −4.6 and +1.6, respectively, disclosing an increase from <5^th^ percentile to the 90^th^ percentile. Thus although the Neuroquant and Neuroreader analyses differed somewhat in the amplitude of the effect detected, they were in agreement that a relatively large magnitude increase in hippo-campal volume had occurred.

Follow-up metabolic analysis also disclosed improvement, with hs-CRP having decreased from 9.9mg/l to 3mg/l, fasting insulin having decreased from 32mIU/l to 8mIU/l, homocysteine having decreased from 15.1μmol/l to 8μmol/l, and 25-hydroxychole-calciferol having increased from 21ng/ml to 40ng/ml. See Table 1 for a summary of the responses of all patients to the treatment program.

**Table 1:** Patient responses to the MEND treatment protocol [3]

Patient	Diagnosis	ApoE Genotype	Treatment Outcome^1^
66yoM	MCI, type 1 (inflammatory)	3/4	Marked subjective improvement, hippocampal volume increase 17^th^->75^th^ %ile
69yoM	AD, type 2 (atrophic)	3/4	Marked subjective improvement, quantitative neuropsychological testing improvement
49yoF	MCI, type 2 (and possibly type 3 (toxic))	4/4	Marked subjective improvement, neuropsychological testing improvement
49yoF	MCI, type 2	2/4	Marked subjective improvement, neuropsychological testing improvement
55yoF	MCI, type 2	4/4	Marked subjective improvement, neuropsychological testing improvement
74yoM	AD, type 1	4/4	Subjective improvement, MMSE 23->30
62yoM	AD, type 1.5 (glycotoxic)	4/4	Subjective improvement, MMSE 22->29
68yoM	MCI, type 1.5	3/4	Subjective improvement, neuropsychological testing improvement
54yoF	AD, type 3	3/3	Clear subjective improvement, MoCA 19->21
54yoF	MCI, type 2	4/4	Subjective improvement, neuropsychological testing improvement

1 See text for details of treatment outcome.

##### Comment

This patient had well documented Alzheimer's disease, with a strongly positive amyloid PET scan, characteristic FDG PET scan, abnormal neuropsychological studies, positive family history, ApoE4-positive (3/4) genotype, and hippocampal volume of 17^th^ percentile. During his 10 months on the MEND protocol, he interrupted his otherwise good compliance once, and this was associated with an episode of memory loss, in which he failed to remember that he had left his car in the driveway while he was working in his house. He returned to the protocol at that time, and after 10 months in total, he demonstrated not only a marked symptomatic improvement (which had begun after approximately three months on the protocol), but also a dramatic increase in hippocampal volume. More modest hippocampal volumetric increases have been described with exercise [9] and with a brain-training program [10], but to our knowledge the magnitude of hippocampal volume increase that occurred with this patient has not been reported previously.

#### Patient 2

This is a follow-up on patient 2 from a previous publication [3]. A 69-year-old entrepreneur and professional man presented with 11 years of slowly progressive memory loss, which had accelerated over the past one to two years. In 2002, at the age of 58, he had been unable to recall the combination of the lock on his locker, and he felt that this was out of the ordinary for him. In 2003, he had an FDG PET scan, which was read as showing a pattern typical for early Alzheimer's disease, with reduced glucose utilization in the parietotemporal cortices bilaterally and left > right temporal lobes, but preserved utilization in the frontal lobes, occipital cortices, and basal ganglia. In 2003, 2007, and 2013, he had quantitative neuropsychological testing, which showed a reduction in CVLT (California Verbal Learning Test), a Stroop color test at 16^th^ percentile, and auditory delayed memory at 13^th^ percentile. In 2013, he was found to be heterozygous for ApoE4 (3/4). He noted that he had progressive difficulty recognizing the faces at work (prosopagnosia), and had to have his assistants prompt him with the daily schedule. He also recalled an event during which he was several chapters into a book before he finally realized that it was a book he had read previously. In addition, he lost an ability he had had for most of his life: the ability to add columns of numbers rapidly in his head.

He was advised that, given his status as an Alzheimer's disease patient and his clear progression, as well as his poor performance on the 2013 test, he should begin to “get his affairs in order.” His business was in the process of being shut down due to his inability to continue work.

His laboratory values included a homocysteine of 18 μmol/l, CRP <0.5mg/l, 25-hydroxycholecalciferol 28ng/ml, hemoglobin A1c 5.4%, serum zinc 78mcg/dl, serum copper 120mcg/dl, copper:zinc ratio of 1.54, ceruloplasmin 25mg/dl, pregnenolone 6ng/dl, testosterone 610ng/dl, albumin:globulin ratio of 1.3, cholesterol 165mg/dl (on atorvastatin), HDL 92mg/dl, LDL 64mg/dl, triglycerides 47mg/dl, AM cortisol 14mcg/dl, free T3 3.02pg/ml, free T4 1.27ng/l, TSH 0.58mIU/l, and BMI 24.9.

He began on the MEND therapeutic program, and after six months, his wife, co-workers, and he all noted improvement. He lost 10 pounds. He was able to recognize faces at work unlike before, was able to remember his daily schedule, and was able to function at work without difficulty. He was also noted to be quicker with his responses. His life-long ability to add columns of numbers rapidly in his head, which he had lost during his progressive cognitive decline, returned. His wife pointed out that, although he had clearly shown improvement, the more striking effect was that he had been accelerating in his decline over the prior year or two, and this had been completely halted.

After 22 months on the program, he returned for follow-up quantitative neuropsychological testing, which revealed marked improvement: his CVLT-IIB had increased from 3^rd^ percentile to 84^th^ percentile (3 standard deviations), total recognized hits from <1^st^ percentile to 50^th^ percentile, CVLT-II from 54^th^ percentile to 96^th^ percentile, auditory delayed memory from 13^th^ percentile to 79^th^ percentile, reverse digit span from 24^th^ percentile to 74^th^ percentile, and processing speed from 93^rd^ percentile to 98^th^ percentile. His business, which had been in the process of termination, was reinvigorated, and a new site was added to the previous sites of operation.

##### Comment

This patient had well-documented Alzheimer's disease, with an ApoE4-positive genotype, characteristic FDG-PET scan, characteristic abnormalities on neuropsychological testing, well documented decline on longitudinal quantitative neuropsychological testing, and progression of symptoms. After two years on the protocol, his symptoms and neuropsychological testing improved markedly. The neuropsychologist who performed and evaluated his testing pointed out that his improvement was beyond that which had been observed in the neuropsychologist's 30 years of practice.

#### Patient 3

A woman late in her fifth decade began to note episodes of forgetfulness, such as returning home from shopping without the items she had purchased. She also placed household items in the wrong locations repeatedly, and frequently failed to recognize previously familiar faces. She had difficulty remembering which side of the road on which to drive. A male cousin had developed Alzheimer's disease in his fifth decade. She was found to be an ApoE4 homozygote. On-line cognitive evaluation showed her to be at the 35^th^ percentile for her age, despite her having been an excellent student earlier in her life.

She began various parts of the MEND protocol, and slowly added protocol features over several months. She began to note improvement, and her on-line cognitive evaluation improved to the 98^th^ percentile, where it has remained to the current time, with her having been on the protocol for 3.5 years.

##### Comment

This patient showed early but definite cognitive decline, documented by on-line quantitative cognitive testing. Her marked improvement has now been sustained for 3.5 years. As described for patient 3 in a previous report [3], her improvement was iterative, with continued optimization over several months.

#### Patient 4

A 49-year-old woman noted progressive difficulty with word finding, and noted that her vocabulary had become more limited. She also began to feel unsure about her navigation during driving. She also complained of difficulty with facial recognition (prosopagnosia). Her recall was affected, and she described the requirement of “more energy” for recall of events. She had difficulty with remembering scheduled events. She also noted that her clarity and sharpness were reduced, leading to difficulties assisting her children with schoolwork. She had difficulty with complex conversations, and with reading comprehension. She also lost the ability she had had to speak two foreign languages.

Her family history was positive for Alzheimer's disease in her father, and her ApoE genotype was 2/4. Her MRI was read as normal, but volumetrics were not included. She underwent quantitative neuropsychological testing at a major university center, and was told that she was in the early stages of cognitive decline and therefore ineligible for the Alzheimer's prevention program, since she was already too late in the disease course for prevention. Her homocysteine was 10μM, hs-CRP 0.6mg/l, hemoglobin A1c 5.2%, fasting insulin 7mIU/l, TSH 1.6mIU/l, and 25-hydroxycholecalciferol 35ng/ml.

She began on the MEND protocol, and over the next several months she noted a clear improvement in recall, reading, navigating, vocabulary, mental clarity, and facial recognition. Her foreign language abilities returned. Nine months after her initial neuro-psychological testing, the testing was repeated at the same university site, and she was told that she no longer showed evidence of cognitive decline. Immediate and delayed recall, as well as semantic knowledge, executive function, and processing speed, had all shown improvement.

##### Comment

This patient had typical early amnestic MCI, which reverted over several months, resulting in a normal neuropsychological examination after nine months. She remains asymptomatic after one year on the program.

#### Patient 5

A 55-year-old woman presented with memory concerns of two-years duration. She had a positive family history of dementia in an aunt and a grandmother. She was an ApoE4 homozygote and a TOMM40 homozygote (G/G).

She experienced difficulties with word recall several times a day, either being unable to recall the word at all or substituting the wrong word in its place. For example, she would say a word like “tweezers” when she meant to say “tongs” (semantic paraphasic errors). She also experienced an increase in spelling errors as she typed on her computer. As a professional writer and editor with a master's degree in English, she found these issues very troubling. She often lost her train of thought while speaking, requiring her to ask others what she had just said. In addition, she would misplace items and forget why she had walked into a room. She would also forget some things her husband had told her or asked her to do.

She began the MEND protocol, and after four months her husband reported that her memory had improved. She noted that her word recall was as good as it had ever been, and she was no longer experiencing an increase in spelling errors. She also reported that she rarely lost her train of thought, but if she went off on a tangent or if someone interrupted her, that issue might return. However, if she paused and gave herself a few seconds, she could find her way back to her original train of thought without asking for help. In addition, she no longer forgot why she had entered a room, and only rarely misplaced items.

Her primary care provider noted that, in her professional opinion, her cognition had returned to normal after four months on the protocol, and an on-line cognitive test (CNS Vital Signs), performed prior to the start of the protocol and then again after five months on the protocol, confirmed this opinion: her overall cognitive assessment (neurocognitive index) had increased from 16^th^ percentile to 73^rd^ percentile; composite memory from 1^st^ percentile to 61^st^ percentile; verbal memory from 3^rd^ percentile to 93^rd^ percentile; visual memory from 5^th^ percentile to 14^th^ percentile; executive function from 14^th^ percentile to 58^th^ percentile; and processing speed from 37^th^ percentile to 81^st^ percentile. Improvement had occurred in all sub-tests.

##### Comment

This patient is homozygous for ApoE4, and presented with amnestic MCI. She showed a clear response, both subjectively and objectively, to the metabolic protocol, and has sustained improvement over seven months.

#### Patient 6

A 74-year-old attorney presented with a five-year history of memory loss and word-finding difficulty. His family history was positive for dementia in his mother, beginning at the age of 75 years. He had been evaluated at an Alzheimer's disease center at the onset of his memory loss, and was found to be ApoE4/4, with MRI showing ventricular enlargement and temporal lobe atrophy, right > left, and FDG-PET showing reduced glucose utilization in the temporal lobes and the precuneus, compatible with Alzheimer's disease. Neuropsychological testing was compatible with a diagnosis of amnestic MCI. He was treated with donepezil, memantine, and intravenous immunoglobulin, and his MMSE fell from 27 to 23 over three years. He noted no improvement with the treatment.

He began the MEND protocol, and after six months, his MFI (phagocytosis index) was measured at 1260, with normal being >500 and most Alzheimer's patients scoring <500 [11, 12]. His MMSE was 29. He returned three months later, his MMSE was 30, and his MFI was 1210. He then returned three months after that, complaining that he had taken a trip, gone off much of the protocol, come under stress, and he felt that his memory had declined. His MFI at that visit had dropped to 230, a typical score for a patient with Alzheimer's disease, and his MMSE was 28. He was placed back on the protocol, and returned two months later, with MFI of 1100 and MMSE of 30. Over the ensuing 12 months, his MFI remained >1000 and his MMSE remained at 30.

##### Comment

This patient, homozygous for ApoE4/4, had a typical amnestic presentation and well documented Alzheimer's disease, unresponsive to donepezil, memantine, and intravenous immunoglobulin. His MMSE improved to a perfect 30 on the metabolic protocol, where it has remained for over one year. His longitudinal MFI supports the notion that MFI may provide a “real time” method for following inflammatory/metabolic status, given the marked reduction when off the protocol with return to normal when he re-initiated the protocol.

#### Patient 7

A 57-year-old man began to have difficulty with memory and in work performance as a computer programmer, leading to dismissal from his job. Over the next five years his cognition continued to decline, he developed navigational difficulties, had difficulty with attention and multi-tasking, and became quieter and less self-assured. He had been a superb guitarist, and he lost both the chord progression memory and the nuance in his playing. Family history was positive for dementia in his mother, in her ninth decade. Evaluation by a neurologist included an unremarkable brain MRI without volumetrics, and he was placed on Aricept, which he discontinued after two months.

Seven years after his symptom onset, he was again evaluated, and found to be homozygous for ApoE4. An FDG PET scan was strongly suggestive of Alzheimer's disease, with reductions in glucose utilization in the temporal, parietal, posterior cingulate, and frontal regions, with some asymmetry. He scored 22/30 on the mini-mental state examination, having lost points for failing to know the date or day, location, and failing tasks of attention and short-term recall. His BMI was 23.

A diagnosis of Alzheimer's disease was made. His laboratory evaluation included an hs-CRP of 0.2mg/l, homocysteine 9.5μmol/l, albumin:globulin ratio of 1.6, hemoglobin A1c 5.7%, fasting insulin 4.9mIU/l, free T3 2.8pg/ml, free T4 1.3ng/l, TSH 2.1mIU/l, testosterone 281ng/dl, pregnenolone 44ng/dl, 25-hydroxychole-calciferol 38ng/ml, total cholesterol 145mg/dl (on atorvastatin), RBC magnesium 4.7mg/dl, serum copper 93mcg/dl, serum zinc 76mcg/dl, copper:zinc ratio 1.22, and AM cortisol 6.8mcg/dl. His Cyrex Array 2 was positive for gastrointestinal hyperpermeability, Cyrex Array 3 (for gluten sensitivity) was negative, and Cyrex Array 20 (for blood-brain barrier disruption) was negative.

He was placed on the MEND protocol, and his MMSE increased to 26 after four months, and to 29 after 10 months. His wife noticed clear improvement in his memory and navigation. His guitar skills improved, both his chord progressions and the nuances of his playing, such that he was able to play several pieces for the neurologist.

##### Comment

This patient had well documented Alzheimer's disease, with a characteristic presentation, characteristic FDG-PET scan, and an ApoE4 homozygous genotype. For the seven years prior to beginning the MEND protocol, his cognition declined, again in keeping with the diagnosis of Alzheimer's disease. Therefore, the chance that his MMSE improved from 22 to 26 and then to 29 over the 10 months on the protocol, as a random event unrelated to the MEND protocol, is slim. Although a score of 29 on the MMSE is within the normal range, both the patient and his wife recognize that subjectively he has not returned completely to normal, and continued optimization of his metabolic status is ongoing.

#### Patient 8

A 68-year-old business executive presented with a five-year history of progressive memory loss, forcing him to retire from his company. He had difficulty navigating while driving, as well. Family history was positive in his mother. He underwent amyloid PET imaging, which was positive. His ApoE genotype was 3/4.

After six months on the MEND protocol, his BMI improved from 27.7 to 24.6, and his hemoglobin A1c improved from 5.9% to 5.7%. Both he and his family noted improvement in memory and navigation. His improvement was documented by on-line neuropsychological testing (Brain HQ), which showed increase from 0 (baseline) to 2221, which represented 52^nd^ percentile for his age.

##### Comment

This patient had typical Alzheimer's disease with mnemonic and visuospatial deficits, progressive course, positive family history, ApoE4 heterozygosity, and a positive amyloid PET scan. He responded to treatment with an improvement in BMI, reduction in hemoglobin A1c, symptomatic improvements in both memory and navigation, and objective improvement in on-line neuropsychological testing.

#### Patient 9

This is a follow-up description of a patient presented in a previous publication [13]. A 50-year-old woman developed depression following a hysterectomy. She received hormone replacement therapy, but the depression continued. At the age of 54, she began to have word-finding difficulty, disorientation, difficulty driving, difficulty following recipes and other instructions, and memory complaints, and these problems progressed. She became quieter and slower to respond. Her depression deepened when her son left home.

She underwent neuropsychological testing, which disclosed frontal, temporal, and parietal abnormalities. A PET scan was typical for Alzheimer's disease, with temporoparietal decreases in glucose utilization as well as a modest frontal decrease. She was placed on duloxetine, which reduced her depression, and donepezil, which improved her cognition. However, she continued to decline.

At the age of 57, she was again evaluated. Her ApoE genotype was 3/3, MoCA was 19/30, BMI was 18, hs-CRP 0.2mg/l, homocysteine 8μM, fasting insulin 4.2uIU/ml, hemoglobin A1c 5.1%, free T3 2.1pg/ml, free T4 1.33ng/dl, reverse T3 23ng/dl, fT3:rT3 9, TSH 1.16uIU/ml, progesterone 0.3ng/ml, AM cortisol 7.2mcg/dl, pregnenolone 19ng/dl, 25-hydroxycholecalciferol 37ng/ml, vitamin B12 799pg/ml, alpha-tocopherol 12.5mg/l, zinc 82mcg/l, copper 99mcg/l, copper:zinc ratio 1.2, ceruloplasmin 20mg/dl, total cholesterol 221mg/dl, HDL cholesterol 67mg/dl, non- HDL cholesterol 167mg/dl, triglycerides 82mg/dl, urinary mercury:creatinine < 2.8, Lyme antibodies negative, C4a 5547ng/ml, TGF-β1 7037pg/ml, and VEGF (vascular endothelial growth factor) 56pg/ml (normal range 31-86pg/ml). VIP (vasoactive intestinal peptide) was not evaluated. HLA-DR/DQ was 13-6-52A (mycotoxin sensitive) and 15-6-51 (Borrelia sensitive). MARCoNS (multiple-antibiotic-resistant coagulase-negative Staph) culture was negative. Anti-thyroglobulin antibodies were strongly positive at 2076IU/ml (normal range 0-0.9IU/ml) and anti-thyroid peroxidase antibodies positive at 58IU/ml (normal range 0-34IU/ml).

She was placed on the MEND protocol, and intranasal VIP (vasoactive intestinal peptide) was administered. After three months, she showed improvement. She was able to babysit her grandchildren. She was able to follow written and verbal instructions without any problems, which had not been possible prior to treatment. She was able to read and remember overnight, and discuss her reading with her husband, which she had not been able to do prior to treatment. She also routinely remembered events of the previous day, which had not occurred in the few years prior to treatment. She had a follow-up MoCA test, and scored 21/30.

##### Comment

This patient had progressed beyond MCI to Alzheimer's disease, well documented by characteristic PET scan abnormalities, neuropsychological testing deficits, and progression. Despite an initial subjective response to donepezil, she continued to decline and displayed significant impairment. She was diagnosed with type 3 Alzheimer's disease [13, 14], and laboratory data supported this diagnosis with characteristic HLA-DR/DQ and abnormal C4a and TGF-β1, as well as anti-thyroglobulin antibodies and anti-thyroid peroxidase antibodies, although MARCoNS culture was negative. After three months of therapy, she showed clear subjective improvement and modest objective improvement. Her previous three years of relentless decline argued against the possibility that the improvement was random and unrelated to her treatment.

#### Patient 10

A 54-year-old woman presented with a two-year history of memory loss. She noted that she did not retain new information the way she formerly had, she had to re-read information a number of times to remember it, especially technical or scientific information, and noted that her reading speed had decreased. She also noted a reduction in vocabulary, word-finding problems, and repeated use of the same word instead of using synonyms. She also noted increased difficulty with grammar and spelling, as well as loss of names of friends and of famous people. Her writing declined, her typographical errors increased, and she had difficulty remembering passwords. She had increasing difficulty driving, organizing, and with her motivation. Activities of daily living were preserved.

Her ApoE genotype was 4/4, homocysteine 7.5μmol/l, hs-CRP 0.26mg/l, albumin:globulin ratio 2.0, hemoglobin A1c 5.3%, fasting insulin 2.7mIU/l, fasting glucose 81mg/dl, alpha-tocopherol 18.3mg/l, and 25-hydroxycholecalciferol 188ng/ml.

On-line quantitative neuropsychological testing disclosed a composite memory score at the 32^nd^ percentile, visual memory at 10^th^ percentile, and verbal memory at 73^rd^ percentile. This testing was repeated after four months on the protocol, at which time the composite memory score was at the 61^st^ percentile, visual memory score at the 25^th^ percentile, and verbal memory score at the 84^th^ percentile.

##### Comment

This person, who is homozygous for the ApoE ε4 allele, demonstrated both subjective and objective evidence of cognitive decline, with preserved activities of daily living, and thus would fit best with a diagnosis of mild cognitive impairment. After four months on the protocol, repeat on-line quantitative neuropsychological testing revealed improvements in visual and verbal memory. Although these improvements were relatively modest, they are in contrast to the natural history of progressive decline in cognition for MCI associated with ApoE4 homozygosity.

## DISCUSSION

These observations provide further support for the previously reported finding that the personalized protocol for metabolic enhancement (note that the metabolic evaluation included parameters shown to affect Alzheimer's disease pathophysiology, such as homocysteine [15], glucose [16], and inflammation [17], as well as numerous others as previously described [3]) in Alzheimer's disease leads to the reversal of cognitive decline in at least some patients with early Alzheimer's disease or its precursors, MCI (mild cognitive impairment) and SCI (subjective cognitive impairment). To our knowledge, the magnitude of the improvements documented in patients 1 and 2 is unequaled in previous reports: in patient 1, the increase in hippocampal volume from 17^th^ percentile to 75^th^ percentile supports the marked symptomatic improvement that he (and others) achieved on the protocol. In patient 2, quantitative neuropsychological testing demonstrated improvements of up to three standard deviations (CVLT-IIB, from 3^rd^ percentile to 84^th^ percentile), with multiple tests all showing marked improvements. These findings complement and support the marked subjective improvement already published for this patient [3].

It is noteworthy that these patients met criteria for Alzheimer's disease or MCI prior to treatment, but failed to meet criteria for either Alzheimer's disease or MCI following treatment—i.e., following treatment, most had returned to the normal range for their cognitive testing. Furthermore, as noted in the initial description of the protocol used here [3], discontinuation of the protocol was associated with cognitive decline (here, in patient 1). It is not yet known for how many months or years the marked improvements will be sustained, but loss of improvement in patients maintaining the protocol has not yet been observed, and follow-ups of up to four years have now occurred.

The hippocampal volumetric increase observed for patient 1 does not discriminate between the possibility that synaptic number increased, or glial cell number or volume increased, or endogenous stem cell survival increased, or neuronal cell number or volume increased, or the vascular compartment increased, or some combination of these possibilities. This volumetric increase, and the marked symptomatic improvement that accompanied it, raises the question of whether it is possible that the patient's diagnosis of mild cognitive impairment associated with Alzheimer's disease was incorrect. However, the diagnostic evaluation makes this possibility extremely unlikely: given the strong family history of dementia, the ApoE4 heterozygosity, markedly positive amyloid PET scan, the FDG-PET scan characteristic of Alzheimer's disease with reduced glucose utilization in a temporoparietal distribution, the abnormal neuropsychological testing, and the MRI showing hippocampal volume at 17^th^ percentile for age, the possibility that the underlying pathological process was something other than Alzheimer's disease is remote. Thus it would be expected that hippocampal volume would decrease over time, and that cognitive decline would occur. Therefore, the likelihood that his improvement was random and unrelated to the intervention is extremely low.

Similarly, for patient 2, it is highly unlikely that the diagnosis of Alzheimer's disease was incorrect: the ApoE4-positive genotype, the FDG-PET scan typical of Alzheimer's disease with temporoparietal reduction in glucose utilization, the pattern and severity of quantitative neuropsychological abnormalities, and the well documented progressive nature of the deficits all provide strong support for the diagnosis of Alzheimer's disease. Furthermore, the severity of the abnormalities documented by the quantitative neuropsychological assessment was also compatible with the diagnosis of Alzheimer's disease. The variations that may occur when different examiners perform the same set of quantitative neuropsychological tests is an obvious concern when there is a significant change in the results of the tests in one subject. However, in this case, the same examiner performed the same set of tests in each instance, arguing against the possibility that the major improvement observed was simply the result of examiner-related variability. The magnitude of the improvement also argued against this possibility.

In each of these cases, obvious subjective improvement, noted by the patient, his/her significant other, and his/her co-workers, was accompanied by clear, quantitated, objective improvement. In the cases of patients 1 and 2, the improvement was of a magnitude not reported previously for patients with Alzheimer's disease. None of the 10 patients exhibited the cognitive decline that is characteristic of Alzheimer's disease, and the improvement experienced by all 10 has been sustained, with the longest time on the program being four years.

It has been claimed that there is nothing that will prevent, delay, or reverse Alzheimer's disease (http://www.nih.gov/news-events/news-releases/independent-panel-finds-insufficient-evidence-support-preventive-measures-alzheimers-disease). Therefore, it is typically recommended that the ApoE genotype, which represents the most important genetic risk factor for Alzheimer's disease, not be evaluated in asymptomatic individuals, and many physicians do not evaluate ApoE genotype even in symptomatic patients. However, the examples described here complement and extend previously published data that argue that these claims are no longer valid. Thus, given the success of the therapeutic regimen used with these patients, it may be appropriate to evaluate the ApoE genotype as part of prevention and early reversal of symptoms. Given the approximately 75 million Americans who are heterozygous for the ApoE ε4 allele, and the approximately seven million Americans who are homozygous, early identification and treatment (presymptomatic or symptomatic) could potentially have a major impact on the prevalence of Alzheimer's disease-mediated cognitive decline.
